# A survey on planar IMRT QA analysis

**DOI:** 10.1120/jacmp.v8i3.2448

**Published:** 2007-07-17

**Authors:** Benjamin E. Nelms, Jeff A. Simon

**Affiliations:** ^1^ Canis Lupus LLC Merrimac Wisconsin; ^2^ Sun Nuclear Corporation Melbourne Florida U.S.A.

**Keywords:** IMRT QA, IMRT, dosimetry, quality system, quality assurance

## Abstract

Quality assurance (QA) systems for intensity‐modulated radiation therapy (IMRT) have become standard tools in modern clinical medical physics departments. However, because formalized industry standards or recommendations from professional societies have yet to be defined, methods of IMRT QA analysis vary from institution to institution. Understanding where matters stand today is an important step toward improving the effectiveness of IMRT QA and developing standards.

We therefore conducted an IMRT QA survey. This particular survey was limited to users of an electronic two‐dimensional diode array device, but we took care to keep the questions as general and useful as possible. The online survey polled institutions (one survey per institution) on a collection of questions about methods of IMRT QA. The topics were general to the IMRT QA analysis methods common to all IMRT systems; none of the questions was vendor‐ or product‐specific.

Survey results showed that a significant proportion of responding institutions (32.8%) use the single‐gantry‐angle composite method for IMRT QA analysis instead of field‐by‐field analysis. Most institutions perform absolute dose comparisons rather than relative dose comparisons, with the 3% criterion being used most often for the percentage difference analysis, and the 3 mm criterion for distance‐to‐agreement analysis. The most prevalent standard for acceptance testing is the combined 3% and 3 mm criteria. A significant percentage of responding institutions report not yet having standard benchmarks for acceptance testing—specifically, 26.6%, 35.3%, and 67.6% had not yet established standard acceptance criteria for prostate, head and neck, and breast IMRT respectively.

This survey helps in understanding how institutions perform IMRT QA analysis today. This understanding will help to move institutions toward more standardized acceptance testing. But before standards are defined, it would be useful to connect the conventional planar QA analyses to their resulting impact on the overall plan, using clinically relevant metrics (such as estimated deviations in dose–volume histograms).

PACS numbers: 87.50.Gi, 87.52.Df, 87.52.Px, 87.53.Dq, 87.53.Tf, 87.53.Kn, 87.56.Fc

## I. INTRODUCTION

With the introduction of intensity‐modulated radiation therapy (IMRT), radiation oncology professionals were required to develop new strategies for stringent quality assurance (QA). Before the advent of IMRT, each radiation field was more or less a customized binary aperture, sometimes used in conjunction with a simple intensity‐modulating device such as a solid or electronic wedge. In this simpler context of three‐dimensional (3D) conformal therapy, the medical physicist could, for example, use proper quality control of the aperture manufacturing process, multileaf collimator (MLC) calibration, and careful modeling of dose estimation by the treatment planning system (TPS) to gain the confidence necessary to approve plan accuracy without the need for QA measurement of each and every beam. But, as is well known, IMRT changed everything.

Each field became a unique “painting” of intensity, optimized for a specific patient's anatomy, beam angle, and planned dose distribution. The dose‐calculation algorithms of the TPSs became more complicated (so as to accurately model the many small and irregular subfields) and more crucial (because of the inherent assumption in inverse planning that the calculation being optimized is accurate in the first place). However, despite the development of new delivery and planning tools, the development of efficient and thorough IMRT QA tools lagged slightly at first, leaving medical physicists to do their work with the limited tools available to them. These new clinical and practical needs created a niche for commercial IMRT QA products, and subsequently a set of IMRT QA systems emerged.

Systems for IMRT QA are now a staple of modern clinics, and they have continued to evolve as the increasing usage of IMRT has precipitated a need for greater efficiency and more advanced features. Over the past few years, medical physicists have shared this set of fairly uniform commercial systems and strategies for IMRT QA. As a result, IMRT programs today almost universally use some method of quantitative comparison between TPS planar dose and measured dose, generating statistics of calculations such as percentage difference, distance to agreement (DTA), and gamma analysis.^(1)^

This first stage of IMRT QA evolution hinged foremost on the wide acquisition and implementation of the new IMRT QA systems. These systems (which include film scanning and calibration, ion chamber arrays, diode arrays, and more recently, megavoltage electronic portal imaging devices) have become regular tools in the modern IMRT clinic. Now, after years of IMRT QA “burn in” time in the real world of busy medical physicists, we can look forward to the next stages of IMRT QA evolution. Three general goals worth considering next would be

- to improve the understanding of current tools and analysis methods, refining them to be as intuitive, efficient, and meaningful as possible;
- to improve existing tools or to develop new tools to continue to meet needs in lockstep with advances in TPSs, delivery, and image‐guided radiation therapy; and
- to propose, prove, and implement universal IMRT QA standards, based on experience and relevant clinical endpoints.

However, before venturing forward on these steps, it would be wise to stop to survey today's methods of IMRT QA, and to develop a realistic understanding of the starting point before strategizing the path to improvement. The present report documents the results of one such IMRT QA survey.

The intent of the present survey was to gather information on these topics:

- Use of single‐gantry‐angle composite (SGAC) exposure as compared with field‐by‐field exposure
- Use of absolute dose as compared with relative dose analysis
- Numeric criteria used in the analysis of percentage difference, DTA, and gamma, plus any lower dose threshold below which statistics are ignored
- Standard acceptance criteria
- Reactions to fields or plans that fail to meet the acceptance criteria

## II. MATERIALS AND METHODS

The goal of the survey was to capture information about the IMRT QA techniques and acceptance criteria used by a population of clinical institutions. To meet this goal effectively, it was important to

- keep the subjects and questions general and vendor‐ or product‐neutral with regard to IMRT TPS, linear accelerator, MLC, or IMRT QA device;
- offer the survey only to clinical medical physicists who perform IMRT QA as part of their regular responsibilities; and
- maintain the anonymity of submitted answers.

The first goal was achieved by framing the topics around the general categories and the tools and techniques that are common to all the major IMRT QA systems. Tables 1 – 4 show the text of the survey questions. It was assumed that each respondent's TPS could produce planar QA dose files (patient IMRT fields calculated separately on a phantom) and that their IMRT QA system could measure the actual planar dose for comparison using industry‐standard methods such as percentage difference, DTA, and gamma analysis. [For clarification, the DTA and gamma methods mentioned here represent slightly different calculations. In DTA, the two‐dimensional (2D) plane is searched radially outward from each measurement point, finding the distance to the nearest point equal in dose (actual or interpolated) on the comparison plane. Gamma is calculated as defined by Low et al.^(1)^]

**Table 1:** Intensity‐modulated radiotherapy quality assurance survey, general methods

Subject/question	Answer options
How often do you use single gantry angle, composite IMRT QA? (i.e. All fields irradiated at normal incidence, added together.)	Never. I always do field‐by‐field analysis.
	Less than 25% of patients
	25–49% of patients
	50–74% of patients
	75–100% patients
Frequency of absolute dose analysis vs. relative dose analysis	I never use absolute dose analysis.(I always use relative dose analysis.)
	I use absolute dose analysis, but less than 50% of the time.
	I use absolute dose analysis approximately 50–74% of the time.
	I use absolute dose analysis approximately 75–99% of the time.
	I use absolute dose analysis 100% of the time.

**Table 2:** Intensity‐modulated radiotherapy quality assurance survey, numeric criteria

Subject/question	Answer options
When generating statistics, my “standard” percent difference (%Diff) analysis level is:	1%
	2%
	3%
	4%
	5%
	>5%
When generating statistics, my “standard” distance‐to‐agreement (mm) analysis level is:	1 mm
	2 mm
	3 mm
	4 mm
	5 mm
	>5 mm
When generating statistics, my “standard” dose threshold (TH) analysis level is:	0 to <5%
	5 to 10%
	≥10%

**Table 3:** Intensity‐modulated radiotherapy quality assurance survey, acceptance criteria

Subject/question	Answer options
Does your clinic have standard acceptance criteria for IMRT prostate fields that are communicated to all applicable employees?	Yes If “Yes” please record: % difference, DTA, gamma criteria Absolute or relative dose
Does your clinic have standard acceptance criteria for IMRT head/neck & brain fields that are communicated to allapplicable employees?	No Not yet, but this is a work in progress.
Does your clinic have standard acceptance criteria for IMRT breast fields that are communicated to all applicable employees?	
Does your clinic have standard acceptance criteria for IMRT SBRT fields that are communicated to all applicable employees?	

**Table 4:** Intensity‐modulated radiotherapy quality assurance survey, reactions to failing quality assurance

Subject/question	Answer options
If the dose analysis for a beam (measured vs. planned) does NOT pass your standard criteria for passing, how do you respond? (You can select more than one.)	I do nothing.
	I do the following: Examine the field(s) and TPS calculated dose to search for known limitations in the IMRT dose algorithm.
	Change the plan if necessary.
	Examine the delivery records or devices (e.g. machine parameters, log files, physical devices, etc.) for errors.
	Change the criteria to get a higher passing rate and record the justification.
	Change the criteria to get a higher passing rate without recording any justification.
	Examine the other beams in the plan to determine if the magnitude of the error might be significant.
	Not applicable, because my analyses always pass the criteria.
	I do something else.

The second and third goals were addressed by the methods of the survey. Inquiry was made to one IMRT QA vendor (Sun Nuclear Corporation, Melbourne, FL) to see if they would help execute the survey. This vendor provides an IMRT QA 2D array measurement and analysis system that has been described previously^(^ ^2^ ^–^ ^3^ ^)^ and that has been adopted by a very large number of institutions. Choosing one vendor with a wide user base was practical for two principal reasons:

- Using multiple vendors might have caused survey respondents (and participating vendors) to be distrustful that the survey might be used indirectly to estimate product market share rather than to focus on IMRT QA methods. Limiting the effort to one vendor avoided this risk.
- Working with one vendor made it easier to secure that vendor's cooperation in using their customer contact channels to announce the survey. This vendor's role was limited to the collection of the survey data and review of the results and manuscript, without the right to disapprove publication.

Of course, limiting the survey to users of one vendor or device introduced some bias into the survey results. For example, only the tools and methods available with that particular device could be surveyed. This consideration was an important one, and care was taken to minimize the effects of these biases. This and other considerations are explored more fully at the beginning of the Discussion section.^b^

Using the vendor's customer database, hundreds of clinical institutions were notified of the survey. The survey questions were posted as an online questionnaire, and the response data were captured electronically, together with institution details and demographics. Each institution was allowed to complete a single, institution‐representative survey response—that is, multiple physicists from one institution could not respond. The IMRT QA response data were stored separately from the demographic and institution data, providing anonymity of the IMRT QA responses.

The goal was to collect responses from at least 100 separate institutions, and the survey was kept “live” online until that number was exceeded. The first reported question in the survey (concerning SGAC exposure as compared with field‐by‐field exposure) was actually a follow‐up question, and it was submitted only to the institutions that responded to the first set of questions. This supplementary question was left online for 1 week.

## III. RESULTS

The goal of collecting responses from 100 or more institutions was easily achieved within a couple of weeks, with a total of 139 institutions choosing to cooperate in the survey. The follow‐up question regarding choice of SGAC or field‐by‐field exposure garnered 64 responses within 1 week when posed to the original 139 responding institutions.

### A. Survey results: general methods

**Figure 1:**
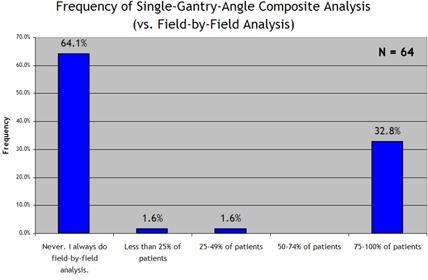
Response to the survey question “How often do you use single gantry angle, composite IMRT QA? (i.e. All fields irradiated at normal incidence, added together.)”

Among the 64 follow‐up respondents, 64.1% said that they use field‐by‐field exposure and analysis all of the time and never use the SGAC technique; 32.8% said that they use SGAC technique for more than 75% of all IMRT plans. Fig. 1 shows the bimodal pattern data, with the responses clustered at both extremes and very few sites reporting a more equal mix of techniques.

**Figure 2:**
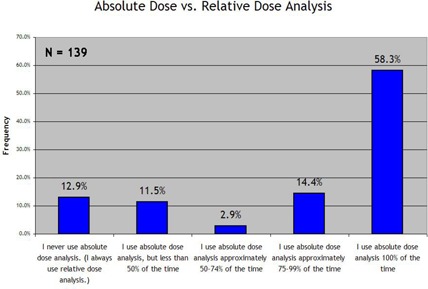
Response to the survey prompt “Frequency of absolute dose analysis vs. relative dose analysis:”

Regarding absolute versus relative dose analysis, 58.3% of the responding institutions use absolute dose 100% of the time. However, a non‐negligible number of institutions never use absolute dose analysis (12.9%) or use it less than half of the time (11.5%), as seen in Fig. 2.

### B. Survey results: numeric criteria for percentage different, DTA, and lower threshold

**Figure 3:**
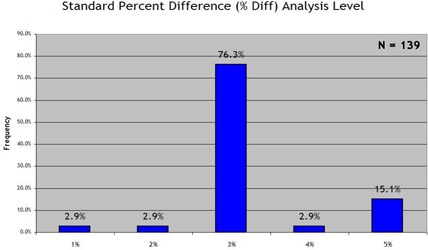
Response to the survey prompt “When generating statistics, my ‘standard’ percent difference (%Diff) analysis level is:”

**Figure 4:**
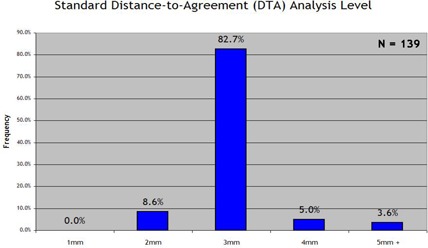
Response to the survey prompt “When generating statistics, my ‘standard’ distance‐to‐agreement (mm) analysis level is:”

**Figure 5:**
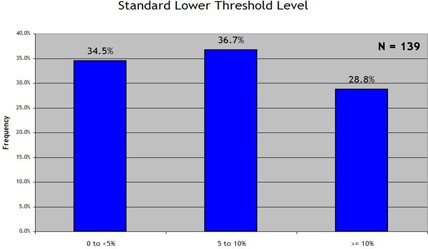
Response to the survey prompt “When generating statistics, my ‘standard’ dose threshold (TH) analysis level is:”

Fig. 3 summarizes the responses on the standard percentage difference criterion, with a 3% difference criterion being reported by a preponderance of institutions (76.3%). As summarized in Fig. 4, an even larger preponderance (82.7%) reported using 3 mm as their standard DTA criterion. Use of the “lower dose threshold”—a dose value defined as the value below which the percentage difference and DTA statistics would be effectively ignored—was much more evenly spread between standard lower dose thresholds of 0%−5%(34.5%), 5%−10%(36.7%), and more than 10% (28.8%), as seen in Fig. 5.

### C. Survey results: acceptance criteria

The questions regarding acceptance criteria were twofold in purpose:

- to determine whether acceptance criteria had been formally established and communicated to all clinicians responsible for IMRT QA, and
- if criteria had been established, to determine what those criteria are.

**Figure 6:**
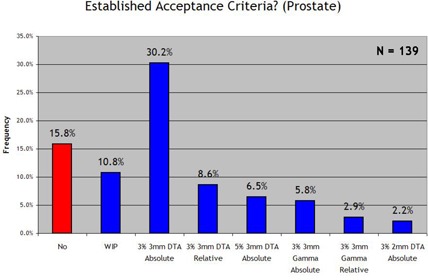
Response to the survey question “Does your clinic have standard acceptance criteria for IMRT prostate fields that are communicated to all applicable employees? (and if so, the criteria)”.

**Figure 7:**
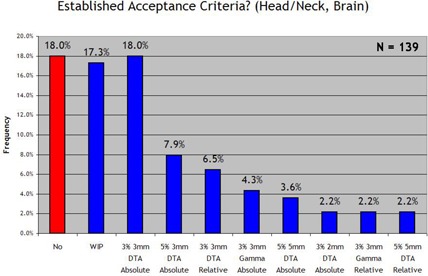
Response to the survey question “Does your clinic have standard acceptance criteria for IMRT head/neck & brain fields that are communicated to all applicable employees? (and if so, the criteria)”.

**Figure 8:**
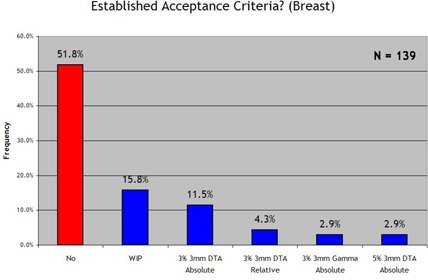
Response to the survey question “Does your clinic have standard acceptance criteria for IMRT breast fields that are communicated to all applicable employees? (and if so, the criteria)”.

**Figure 9:**
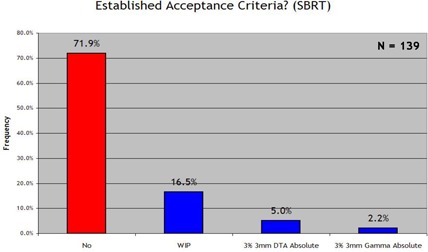
Response to the survey question “Does your clinic have standard acceptance criteria for IMRT SBRT fields that are communicated to all applicable employees? (and if so, the criteria)”.

We examined four general anatomic disease categories separately [prostate, head and neck and brain, breast, and stereotactic body radiotherapy (SBRT)]. Figs. 6–9 show the results. With regard to prostate IMRT, 26.6% of the responding clinics had not yet established acceptance criteria for IMRT QA. For IMRT of the head and neck and brain, 35.3% of the institutions had not yet established standard acceptance criteria. For breast plans and lung or SBRT plans, in which IMRT is less frequently used, little progress was seen in the way of establishing IMRT QA acceptance criteria for the two disease sites: 67.6% and 78.4% of all responding institutions had not yet set standards for breast or SBRT IMRT respectively. When an institution reported that it had established acceptance criteria, it most often used the 3% and 3 mm DTA absolute dose analysis, which was the most common method used in each anatomic site category. When institutions had established acceptance criteria, the number of points required to “pass” the 3%/3 mm DTA combined analysis was generally 90%−95%.

### D. Survey results: reactions to failing fields

Table 5 shows the frequency of common actions taken in response to failing fields. The most common reaction, used by 72.1% of respondents, was to examine the TPS‐calculated dose planes to probe for deviations caused by known limitations in the dose calculation algorithm. A reaction used by 61.2% of the respondents was to examine the errors in light of the other beams in the plan to try to qualify the overall significance of the errors. About half (51.8%) reported that they proactively change the plan if necessary to achieve acceptable IMRT QA analysis statistics.

**Table 5:** Reactions to fields that fail to meet acceptance criteria, multiple responses accepted

Frequency of responses to the question “If the dose analysis for a beam (measured vs. planned) does NOT pass your standard criteria for passing, how do you respond?”	
Response	Response frequency (% of N=139)
Examine the field(s) and TPS calculated dose to search for known limitations in the IMRT dose algorithm	71.2%
Examine the other beams in the plan to determine if the magnitude of the error might be significant	61.2%
Change the plan if necessary	51.8%
Examine the delivery records or devices (e.g. machine parameters, log files, physical devices, etc.) for errors	42.4%
Change the criteria to get a higher passing rate and record the justification	39.6%
Change the criteria to get a higher passing rate without recording any justification	6.5%
Not applicable, because my analyses always pass the criteria	2.9%
I do nothing	0.0%
I do something else	59.0%

In addition to the common actions that were tallied, many “custom” responses were submitted to this survey question. The most common category of custom response involved use of a micro‐chamber to verify absolute dose on the central axis or away from steep gradients. Four custom responses mentioned that they might use film to repeat the QA and inspect the high‐gradient regions. One particular custom response was very unique and warrants attention: “We have a statistical database of % passing and other measures. Aparticular set of measures is related to this database, resulting in a % rank. These % ranks are then related to qualitative values such as ‘excellent’ or ‘good’ etc. as to how the current set of data relates to the [sic] history of QA for a particular treatment area (prostate, head and neck, etc). The physician is presented with the final analysis and makes the final decision about whether to accept the results.”

## IV. DISCUSSION

### A. Discussion of potential survey biases

Any survey should acknowledge potential sources of bias in the data. Three major potential sources of bias must be acknowledged in the present work.

First, this survey was posed to a population of users of a specific 2D diode array product. Great care was taken to make the questions generic to any IMRT QA system, but of course clinical methods often correlate with the available tools and the completeness of the users' training on those tools. As mentioned earlier, it would be useful for other independent researchers to conduct similar surveys with other prominent IMRT QA systems, sampling their user bases as new populations.

Second, this survey was purely voluntary—a situation that lends itself to natural biases based on who is likely to respond versus who is likely not to respond. We can hypothesize about such potential biases, such as a higher likelihood of experienced IMRT QA users responding as compared with those who are less experienced or less interested in process improvement; however, this conjecture cannot be proven. It is worth mentioning that the spirit of this work was to catalyze an understanding of where IMRT QA is today so that methods and processes can realistically be improved in future. In this regard, a bias toward those more experienced in IMRT QA would not necessarily be bad.

Third, the survey results presented here were weighted by institution and not by individual medical physicist—that is, an institution with 1 medical physicist treating 10 IMRT patients daily was given the same weight in the data analysis as an institution with 5 medical physicists treating 100 IMRT patients daily. Again, this choice was made because the intent was that the survey should shed light on IMRT QA standards between institutions so as to incite discussion on industry‐wide expectations and standardization.

In addition, this survey was limited to planar dose analysis for normally incident IMRT beams and did not query the method of SGAC IMRT QA (discussed further below).

### B. SGAC as compared with field‐by‐field IMRT QA

Obviously, a substantial percentage of institutions (32.8%) use the SGAC technique for most IMRT QA procedures. In the SGAC technique, all fields in a plan are delivered to a flat phantom at coinciding normal incidence, and the sum of all the fields is compared with an analogous plane for which all beams are calculated and summed by the TPS.^c^ The assumption had been that the SGAC method, a vestigial remnant of the days of laborious film dosimetry, was rarely used any more, considering that irradiating and processing a single film instead of many films could save significant time. Electronic 2D arrays enable field‐by‐field IMRT QA to be performed more efficiently, but our survey suggests that field‐by‐field analyses have not completely replaced the SGAC method.

The efficacy of SGAC as compared with field‐by‐field analysis in IMRT QA would benefit from a separate study. The SGAC technique sums into one plane the critical areas of high dose, low dose, and steep gradients from each IMRT field, potentially masking the complex structure of the individual fields and making detection of errors in delivery or TPS calculations more difficult. In fact, the true clinical significance of the errors detected with either SGAC or field‐by‐field IMRT QA is not well understood. It would be beneficial for experienced IMRT institutions to perform research on these IMRT QA methods, quantifying their respective abilities to detect and diagnose errors, and proposing how those errors might be translated into more clinically relevant conclusions.

### C. Absolute compared with relative dose

The survey results on the use of absolute dose versus relative dose comparisons show that absolute dose analysis is more prevalent. More than half (58.3%) of respondents analyze absolute dose exclusively, with almost three‐quarters (72.7%) using absolute dose analysis 75% or more of the time. These results are encouraging, because analyzing relative dose leaves open the possibility of global and significant errors in the overall dose level; analyzing absolute dose combines analysis of intensity modulation with analysis of dose output (and therefore monitor unit) accuracy.

### D. Analysis and acceptance criteria

An obvious result of the survey is that the “3%/3 mm DTA” method represents the dominant choice for generating pass/fail statistics. To date, no known study qualifies (or disqualifies) these criteria as an apt “gold standard” for planar dose to phantom IMRT QA, based on any resulting estimated deviations in the 3D dose to the patient.

Some follow‐up is relevant. Why are these acceptance criteria so prevalent, and are they proven to be adequate? Put another way, if every field has 95% of its points within 3% and 3 mm using absolute dose analysis, would the resultant DVH curves (inheriting all the deviations between actual fields and planned fields) also be acceptably close to the plan DVH curves? In particular, the 3 mm DTA criterion is fairly liberal when it comes to complex IMRT fields with steep gradients.

It is also important to realize that a common method of calculating percentage difference^(4)^ uses a single normalizing dose value as the denominator of the percentage calculation. This approach is intended to keep the percentage difference calculation normalized to a relevant prescribed dose. For example, if the normalizing dose for a whole composite fraction is 2 Gy, then a 1% difference means a dose difference of 0.02 Gy, regardless of the local dose. This method of calculating percentage difference is useful when analyzing true composite dosimetry (that is, actual 3D treatment angles, applicable phantom); however, it is less obvious when analyzing single modulated fields that, taken as a single entity, lack a normalizing or prescribed dose. The alternative is always to use the local dose as the denominator of the percentage difference calculation; however, that approach leads to inflated percentage difference calculations at low dose levels. For example, calculated based on local dose, the 0.02 Gy difference between 0.22 Gy measured and 0.20 Gy calculated yields a 10% percentage difference. It is important for a medical physicist to completely understand how these important criteria are calculated before adopting a standard.

Our survey shows that a significant percentage of institutions have not yet established institution‐wide IMRT QA acceptance criteria. To ensure that IMRT QA serves tangible purposes (for example, error detection and diagnosis, patient safety, institution risk management) and is not just a task to be performed and documented, defined processes and benchmarks are needed to justify the acceptance of passing plans and remediation of failing plans. Defining such processes and benchmarks requires not only a focused effort, but perhaps a new—or at least revised—approach to IMRT QA analysis. Notably, the very procedural mindset illustrated by the responding institution that designed a “ranking” procedure in light of a historical database of results is the type of progressive and proactive mindset that needs to be adopted as technology moves forward. To progress to the next stage of IMRT QA, IMRT QA analysis tools are needed that provide more clinically relevant output so that each plan can be ranked according to intuitive and justifiable benchmarks.

### E. Looking forward

The underlying limitation of today's planar IMRT QA approach is that it does not make the connection between the individual field analyses and the “big picture” of how the patient dose distribution might be affected—that is, how the plan DVHs might be degraded as a result of the combined planning and delivery imperfections. Today, the DVH is the critical tool for IMRT dose prescription and plan analysis. An estimated DVH (based on measurements) should perhaps be the new goal of IMRT QA. Although careful field‐by‐field analyses are now efficient and very effective at detecting differences between the measured fields and the planned fields, they do not predict the overall perturbations of the volumetric patient dose and DVH statistics. If meaningful standards for IMRT QA acceptance testing are to be derived and adopted, that connection needs to be made.

Estimating DVH perturbations attributable to IMRT QA measurements would be a wise first step in trying to introduce meaningful standards to IMRT QA, because the benchmarks could be set based on more clinically relevant and intuitive endpoints. One strategy to recalculate the plan dose based on an estimation or derivation of actual delivered fluence and then to compare the original and the newly calculated DVH statistics has been proposed.^(5)^ Although that method is complicated by its introduction of yet another independent 3D dose calculation algorithm (which will invariably have its own inherent limitations), its intent is a step in the right direction.

The development by IMRT QA vendors of methods or algorithms to compare measurement‐derived, estimated DVHs against planned DVHs would perhaps be the greatest step towards establishing clinically relevant IMRT QA standards. With such standards in place, remediation of plans that fail to pass the criteria could be proactive and purposeful. That is, remediation would mean eliminating enough error that IMRT QA testing meets clinically relevant standards. The common reactions that medical physicists use today, such as those presented in Table 5, could evolve to be actions that eliminate sources of error to meet a benchmark rather than primarily to explain and document the errors. That is the essence of any quality system, and progress in that direction can be achieved with a coordinated effort by clinical medical physicists and IMRT QA vendors.

## V. CONCLUSIONS

We conducted a survey across many clinics (N=139) of the methods used for IMRT QA analysis (measured vs. calculated dose planes). The major conclusions of our survey were these:

- A significant fraction of the responding institutions (32.8%) still use a SGAC method instead of a field‐by‐field method for most IMRT QA analyses.
- More than half of the institutions perform absolute dose comparisons rather than relative dose comparisons.
- The 3% criterion is the one most used for percentage difference analysis, and the 3 mm criterion for DTA analysis, with the most prevalent standard for acceptance testing being the combined 3%/3 mm criteria.
- A significant percentage of institutions have not yet established standard benchmarks for acceptance testing (26.6%, 35.3%, and 67.6% of respondents had not yet set standard acceptance criteria for prostate, head and neck and brain, and breast IMRT respectively).

Therefore, even though the tools for performing IMRT QA have become common, each with a fairly standard set of features, the acceptance criteria vary from one institution to another or have yet to be formally defined at all. If standardization of IMRT QA analysis and acceptance testing is made a goal, the first need will be to connect the analysis of planar QA accuracy to the resulting impact on clinically relevant plan metrics. To this end, estimation of DVH effects would be one promising strategy in the future of IMRT QA.

## ACKNOWLEDGMENTS

We are very appreciative of the time taken by all the medical physicists who responded to the survey. The sharing of your experiences and methods will help in the continual improvement of IMRT QA. We also thank Sanjeev Saini of Sun Nuclear Corporation for coding and posting the online survey and assembling the response data. This work was funded in part by Sun Nuclear Corporation.

## Footnotes

b We encourage other independent authors to cooperate with other IMRT QA vendors on the same type of project to determine if product‐specific biases appear in the survey results.

c This approach is not to be confused with the true method of composite IMRT QA, in which a cylindrical phantom is exposed to all IMRT beams at their actual treatment orientations, followed by comparison of the axial, coronal, and/or sagittal planes of the relevant geometric composite dose with calculation.
